# Measuring quality of life in autistic children and young people: comparing the performance of common generic health related quality of life instruments

**DOI:** 10.1007/s11136-026-04246-4

**Published:** 2026-06-05

**Authors:** Lee Constable, Rachel O’Loughlin, Renee Jones, Harriet Hiscock, Kim Dalziel, Quality of Life in Kids: Key evidence to strengthen decisions in Australia (QUOKKA) project team, Richard Norman, Rosalie Viney, Julie Ratcliffe

**Affiliations:** 1 Health Services and Economics, Murdoch Children’s Research Institute, Parkville, VIC 3052 Australia; 2 Melbourne Health Economics, Centre for Health Policy, Melbourne School of Population and Global Health, University of Melbourne, Melbourne, VIC 3010 Australia; 3 Department of Paediatrics, Melbourne Medical School, University of Melbourne, Melbourne, VIC 3010 Australia

**Keywords:** Health related quality of life, Autism spectrum disorder, Child, Adolescent, Health care

## Abstract

**Purpose:**

To assess the comparative measurement performance of commonly used paediatric health related quality of life (HRQoL) instruments in autistic children.

**Methods:**

The sample was 510 autistic children aged 5–18 years from the Australian Paediatric Multi-Instrument Comparison study. The psychometric performance of the PedsQL, EQ-5D-Y-5L, EQ-5D-Y-3L, CHU9D and KIDSCREEN-27 instruments was compared. Psychometric attributes assessed included acceptability (ease/difficulty completing), feasibility (time to complete), test-retest reliability, floor/ ceiling effects, convergent validity and known-groups validity (measured through schooling, special health care needs (SHCN), and the strengths and difficulties questionnaire (SDQ)). Sub-group analysis by reporter type (child vs. caregiver), child age (5–12 vs. 13–18 years) and child gender (male vs. female) was conducted.

**Results:**

All measures demonstrated similarly good acceptability, feasibility, and convergent validity. Only the PedsQL, EQ-5D-Y-5L and EQ-5D-Y-3L demonstrated test-retest reliability (intraclass correlation coefficient 0.56–0.60). No floor or ceiling effects were found in the total sample however, ceiling effects for the EQ-5D-Y-3L were noted in 13–18 year olds (19.1%), males (15.6%), and children who self-reported (15.0%). For known-groups validity (Cohen’s *d* > 0.5), only the EQ-5D-Y-5L differentiated between children in mainstream and specialist schools, only the CHU9D and PedsQL differentiated between children with and without SHCNs, and all measures differentiated between children with and without behavioural difficulties as per the SDQ.

**Conclusion:**

The PedsQL and EQ-5D-Y-5L were the strongest performing measures in autistic children. As performance varied by reporter type, child age, and child gender, those selecting a measure should assess evidence in their intended population of use.

**Supplementary Information:**

The online version contains supplementary material available at 10.1007/s11136-026-04246-4.

## Abstract (plain language summary)

Globally, 1 in every 100 children is diagnosed with Autism Spectrum Disorder (ASD). ASD is known to have large impacts on a child’s life, impacting their quality of life and wellbeing. It is essential to accurately measure the quality of life of autistic children, as this can help understand if treatments, services and other things are helping to improve their wellbeing. Although we have a range of different quality of life questionnaires we can use in children, we don’t know how well these quality of life questionnaires perform in autistic children. The purpose of this study is to compare different quality of life questionnaires in autistic children to see which questionnaire would be most appropriate for measuring their quality of life.

**Supplementary Information:**

The online version contains supplementary material available at 10.1007/s11136-026-04246-4.

## Background

Autism Spectrum Disorder (ASD) is a lifelong developmental condition characterised by challenges with social communication and restricted, repetitive patterns of behaviour, interests, or activities, and sensory processing challenges [1]. Globally, the prevalence of children with ASD is approximately 1% [2], whilst in Australia, the prevalence is estimated to be 2–5% [3]. ASD also has one of the highest burdens on children, with the 2023 Australian Burden of Disease Study identifying ASD as the leading cause of burden in males, and fourth leading cause of burden in females aged 5–14 years [4]. ASD is also known to be associated with poorer health-related quality of life (HRQoL) in children. HRQoL is an indicator of overall health and considers both physical and mental health and their impacts on an individual’s functioning, as rated by the child themselves, or a proxy such as a parent [5].

Children’s HRQoL can be assessed via condition-specific or generic HRQoL instruments. Condition-specific instruments allow in-depth examination of factors that affect health that are unique to that health condition. No condition-specific HRQoL instruments exist for children with ASD. On the other hand, generic HRQoL instruments have many uses and benefits. In routine clinical care, generic HRQoL instruments can be a way to engage in more patient centred care [6], in population health research they can be used to understand and compare the HRQoL of populations [7], and finally, in health economics they can be used to inform health technology assessment, economic evaluations, and subsequent health policy making [8]. Generic HRQoL instruments are particularly useful in these contexts as they provide broad information on patient’s HRQoL, with the advantage of allowing comparison between different health conditions, treatments and populations [7, 9]. Using generic HRQoL instruments in autistic children for the aforementioned uses could be beneficial in informing care and priorities setting, however, it requires valid estimates of HRQoL in autistic children. This is challenging as little is known of the validity and reliability of generic HRQoL instruments in autistic children. Three recent systematic reviews [10–12] synthesised the existing evidence on the psychometric properties of a range of generic HRQoL instruments for paediatric populations. Of these, only one (Rowan et al. 2021) included a study specific to autistic children that assessed the known groups and convergent validity of the Health Utilities Index Mark III (HUI3) [10, 13]. In addition to these reviews, three other studies have been published that assess the performance of generic HRQoL measures in autistic children [14–16]. One examined the construct validity of the HUI3 and the Quality of Well-Being Self-Administered (QWB-SA) instruments [14]. Another examined acceptability, reliability, validity, and responsiveness of the Pediatric Quality of Life Inventory 4.0 generic core (PedsQL) and CHU9D instruments [15]. Finally, one evaluated construct validity and responsiveness of the CHU9D and EQ-5D-Y [16]. All studies have been in children aged 10 years or older. This current evidence base precludes comparisons across common generic HRQoL measures as studies only included two measures and assessed different psychometric properties. Furthermore, no studies assessed the performance of generic HRQoL instruments in younger autistic children (aged 5–9) and no studies have examined psychometric properties of the instruments in different sub-populations of interest, such as by report type (self- vs. proxy-report) or by gender, despite evidence that differences arise for these subgroups in other conditions and populations [17].

To address these limitations in the evidence base, this study aims to assess the acceptability, feasibility, reliability, floor and ceiling effects, convergent validity and known groups validity across a comprehensive range of commonly used generic HRQoL instruments in autistic children. Subgroups are analysed based on reporter type, child age and child gender. This information can inform clinical and health economic decision making for appropriateness of different HRQoL instruments for use with autistic children.

## Methods

### Study design

#### Participants

Data were from the existing Australian Paediatric Multi-Instrument Comparison (P-MIC) Study [18, 19]. The P-MIC study was conducted between November 2021 and August 2022, with participants with certain health conditions, such as ASD, recruited via an Australian online panel survey company (Pureprofile Pty Ltd Australia) [20]. Only children recruited into the specific ASD cohort (caregiver report of any child aged 5–18 years with ASD diagnosed by a health professional) for the P-MIC study were used in this analysis [20]. The screening question used to determine inclusion criteria was adapted from the Longitudinal Study of Australian Children (LSAC) [21].

#### Data collection

Study participants completed an initial survey, including demographic questions, the Children with Special Health Care Needs (SHCN) Screener, the Strengths and Difficulties Questionnaire (SDQ); and five paediatric generic HRQoL instruments (PedsQL, EQ-5D-Y-5L, EQ-5D-Y-3L, CHU9D and KIDSCREEN-27). Four weeks after the initial survey, all participants were asked to complete a follow-up survey where they repeated four of the generic HRQoL instruments (all except KIDSCREEN-27 due to lived experience feedback on follow up survey length). In the follow-up survey, participants were also asked about any change in the child’s health status (see Supplementary Table S1). All instruments were completed as an online survey, with the initial survey designed to take 15–30 min, and the follow-up survey designed to take approximately 5 min to complete. The children were able to complete the HRQoL instruments portion of the survey (self-report) if they were over 7 years of age and their caregiver considered them capable of completing the instruments. Otherwise, all instruments were completed by the caregiver (proxy-reported) [18]. All participants were required to complete all questions within each instrument, therefore there were no missing data [18].

#### Instruments

The five paediatric HRQoL instruments were prioritised from the P-MIC study as they are either preference weighted (EQ-5D-Y-3L, CHU9D) or have imminent preference weighting available (PedsQL, EQ-5D-Y-5L. The PedsQL, EQ-5D-Y-5L, EQ-5D-Y-3L and CHU9D were included as commonly used generic HRQoL instruments [10, 20]. The KIDSCREEN was selected in the P-MIC as the most validated HRQoL measure for use with autistic children, given no condition-specific HRQoL instrument exists. The KIDSCREEN-27 was included as it had previously been suggested as an appropriate instrument for use in this population [20, 22, 23] due to its unique framing of the questions with a positive strengths-based language, in contrast to the deficits-based language used in other instruments. This aligns with the ethos of the autistic community and the Convention on the Rights of Persons with a Disability (CRPD) [24]. Table 1, below, provides a summary of each instrument.

**Table 1:** HRQoL Instrument Description and Summary

Instrument	No. of items	Item response levels	Report type	Age range	Recall period	Domains	Total score or LSS range*	Better HRQoL Higher/Lower scores
Pediatric Quality of Life Inventory 4.0 generic core PedsQL [43]	23	5	Proxy & Self	8–12y &13–18y	1 month	Physical, emotional, social and school functioning	0–100	Higher
EQ-5D-Y-5L [19, 44, 45]	5	5	Proxy & Self		Today	Mobility, looking after self, usual activities, pain or discomfort, and worried or sad	5–25	Lower
EQ-5D-Y-3L [19, 44, 45]	5	3	Proxy & Self		Today	Mobility, looking after self, usual activities, pain or discomfort, and worried or sad	5–15	Lower
CHU9D [46]	9	5	Proxy & Self		Today	Worried, sad, pain, tired, annoyed, schoolwork or homework, daily routine, activities, and sleep	9–45	Lower
Kidscreen-27 [22, 23]	27	5	Proxy & Self		1 week	Physical wellbeing, psychological wellbeing, autonomy and parent relations, peers and social support, school environment	27–135	Higher

*Total score (as per the instrument scoring algorithm): PedsQL, Kidscreen-27; Level sum score (LSS) (calculated by adding the numerical values for each item response together to form a total):

EQ-5D-Y-5L, EQ-5D-Y-3L, CHU9D [19, 46].

Instrument total scores or level sum scores (LSSs) were used in the main analysis in place of the typical utility score to enable a comparison with the PedsQL, EQ-5D-Y-5L and KIDSCREEN-27 which do not have accompanying value sets. Utility scores for use in sensitivity analyses were calculated for the EQ-5D-Y-3L and CHU9D using appropriate utility scoring algorithms [25, 26].

The SDQ is a 25-item commonly used screening measure of emotional and behavioural difficulties in children and young people [27]. The SDQ was included to enable comparison of performance of HRQoL instruments against a validated scale of emotional wellbeing [20].

### Psychometric analyses

All analyses were conducted using Stata Version 18 (Statacorp, College Station, TX, USA).

#### Acceptability

Acceptability was measured by self-reported difficulty completing each of the instruments. Participants were asked to rate difficulty completing each instrument on a 5-point scale from 1) very difficult to 5) very easy. Acceptability data were assessed descriptively.

#### Feasibility

Feasibility was measured by the time to complete each of the instruments within the initial survey. This was captured automatically by REDCap, the online platform used to administer the survey [28]. Times greater than 10 min per instrument were deemed likely as a result of people leaving the survey open while partially complete while not attending to the task and were top-coded as per previous similar studies [29]. As data were positively skewed, median times and their corresponding inter-quartile range (IQR) are reported.

#### Test-retest reliability

Test-retest reliability was assessed using intra-class correlation coefficients (ICCs) and corresponding 95% confidence intervals. ICC was calculated using a two-way mixed-effects model for a single instrument, based on absolute agreement [20]. ICC thresholds based on Koo et al. were used for interpretation with < 0.5 indicating poor, 0.50–0.74 moderate, 0.75–0.90 good, and > 0.90 excellent reliability [20].

Test-retest reliability measures the ability of an instrument to provide stable scores on repeated measurement, provided there is no change in health status. Therefore, these analyses *excluded* observations where the participant reported a change (either an improvement or a worsening) in the child’s health status between the initial and follow up survey.

#### Floor and ceiling effects

Floor and ceiling effects are indicated when a high proportion of respondents are at the extreme upper or lower end of scores for an instrument and indicate that an instrument is not effectively reflecting a wide range of possible health states [20]. An instrument was considered to have a ceiling effect if ≥ 15% participants reported the lowest severity level (i.e. best possible HRQoL) for all items. An instrument was considered to have a floor effect if ≥ 15% participants reported the highest severity level (i.e. poorest possible HRQoL) for all instrument items [30, 31].

#### Convergent validity

Convergent validity was used to measure the extent to which the instruments were correlated in line with hypotheses. Typically, convergent validity is assessed against a gold standard instrument however, there is no gold standard instrument in this population. In this case, we assessed convergent validity as the correlation between the PedsQL and all other HRQoL instruments, as this approach has been used previously given PedsQL is commonly used in clinical care in this population [17, 19]. Correlations were assessed using Spearman’s correlations due to non-normally distributed data [20]. Correlations were considered weak between 0.1 and 0.29, moderate between 0.3 and 0.49, and strong at ≥ 0.5 [32].

As all the instruments are designed to examine the construct of HRQoL, we hypothesised the EQ-5D-Y-5L, EQ-5D-Y-3L, CHU9D would be strongly correlated with the PedsQL. The KIDSCREEN-27, due to the strength-based framing and inclusion of the domain ‘autonomy and parent relations’ unique to this instrument, was hypothesised to have a moderate correlation.

#### Known-groups validity

Known-groups validity assesses the ability of an instrument to detect differences in HRQoL between two groups, where such a difference is known or hypothesised to exist. Known-group validity was assessed for three groups:

(i) ASD severity (children attending mainstream vs. specialist school): As a proxy for clinically diagnosed severity, ASD severity was approximated using the school setting the child attended: a ‘specialist school’ designated for those with disability or high needs, or ‘mainstream school (either with or without integration support funding to support additional needs)’. Participant data were excluded from this analysis only if the child was not attending school.
(ii) Special healthcare needs (SHCN) (children without vs. with a SHCN): SHCN was determined based on a brief, validated, screener which asked if the “*Child has a condition which has lasted or is expected to last for at least 12 months which causes them to use medicine prescribed by a doctor (other than vitamins) or more medical care*,* mental health or educational services. Yes/no*” [33].
(iii)  SDQ scores (normal vs. ‘abnormal’ or ‘of concern’ behavioural difficulties): SDQ score thresholds were determined based on validated Australian population norms based on the child’s age and gender. Though these were calculated individually for each child, generally, ‘abnormal’, or ‘of concern’, was considered for scores > 15–17 of a total possible score of 40 [34, 35].

We hypothesised that HRQoL would be poorer for those with greater ASD severity (i.e., attending specialist school), presence of SHCN and higher SDQ scores (i.e., ‘abnormal’ or ‘of concern’ behavioural difficulties).

Instruments were considered to demonstrate known groups validity if independent *t*-tests resulted in a *p* value < 0.05 and at least a moderate effect size (> 0.5). Effect sizes (ES) were estimated using Cohen’s *d* with an effect size 0.2–0.49 considered small, 0.5–0.79 considered moderate and ≥ 0.8 considered large [32].

#### Summary of instrument performance

A summary table of psychometric properties with validated thresholds (test-retest reliability, ceiling effects, convergent validity and known groups validity) of all instruments was created as a high-level overview of performance. For each of the psychometric analyses, results were categorised into three levels of performance. See footnotes of Table 3 for details.

Whilst analysis of groups with sample sizes of < 50 was conducted, they are excluded from the summary of instrument performance (and marked as ‘?’) as per the COSMIN guidelines which state that sample sizes under 50 are considered ‘inadequate’ or ‘doubtful’ [36].

#### Subgroup and sensitivity analyses

All psychometric analyses were repeated for each subgroup of interest: (i) Report type (self vs. proxy), (ii) Child age (5–12 vs. 13–18 years old) and (iii) Child gender (male vs. female). Children who identified as a gender other than male or female were excluded from the child gender subgroup analysis due to low sample size. These sub-groups are based on those suggested by the P-MIC technical methods paper [20].

Sensitivity analysis was performed, where feasible, using utility scores rather than total scores/LSS. Analysis was performed on test-retest reliability and known-group validity for the EQ-5D-Y-3 L and CHU9D using available value sets [25, 26].

## Results

### Sample characteristics

Sample characteristics are reported in Table 2. The sample comprised 510 respondents. Approximately two thirds self-completed the HRQoL instruments (64.1%). Caregivers were predominately female (83.7%) and 26.3% had a bachelor’s degree or higher. Most children were male (70.2%) and overall child mean age was 10.6 years old (range = 5–18 years old).

**Table 2:** ASD Sample characteristics by report type, child age and child gender

Characteristic	Total ASD Sample *n* (%)	Report type		Child age		Child gender	
		Proxy *n* (%)	Self *n* (%)	5–12 years *n* (%)	13–18 years *n* (%)	Male *n* (%)	Female *n* (%)
Respondents	510 (100)	183 (35.9)	327 (64.1)	337 (66.1)	173 (33.9)	358 (71.9)	140 (28.1)
Caregiver Age, mean (SD)	40.1 (8.5)^#^	39.6 (9.3)	40.4 (8.1)	37.7 (7.8)	44.9 (7.9)	40.0 (8.2)	40.1 (9.5)
Caregiver Gender							
Male	82 (16.1)	34 (18.6)	48 (14.7)	59 (17.5)	23 (13.3)	57 (15.9)	25 (17.9)
Female	427 (83.7)	149 (81.4)	278 (85.0)	278 (82.5)	149 (86.1)	301 (84.1)	115 (82.1)
Transgender male							
Not described	1 (0.2)	0 (0.0)	1 (0.3)	0 (0.0)	1 (0.6)	0 (0.0)	0 (0.0)
Caregiver Education level							
Bachelor degree or higher	134 (26.3)	49 (26.8)	85 (26.0)	92 (27.3)	42 (24.3)	92 (25.7)	39 (27.9)
Tertiles of SEIFA IRSAD^#^							
Low	227 (44.8)	80 (44.0)	147 (45.2)	154 (45.8)	73 (42.7)	154 (43.3)	65 (46.8)
Middle	160 (31.6)	56 (30.8)	104 (32.0)	102 (30.4)	58 (33.9)	114 (32.0)	44 (31.7)
High	120 (23.7)	46 (25.3)	74 (22.8)	80 (23.8)	40 (23.4)	88 (24.7)	30 (21.6)
Child Aboriginal and/or Torres Strait Islander origin	46 (9.0)	15 (8.2)	31 (9.5)	32 (9.5)	14 (8.1)	35 (9.8)	11 (7.9)
Child Language other than English spoken at home	23 (4.5)	10 (5.5)	13 (4.0)	15 (4.5)	8 (4.6)	18 (5.0)	4 (2.9)
Child Age, mean (SD)	10.6 (3.7)	9.2 (4.1)	11.4 (3.2)	8.3 (2.2)	15.0 (1.6)	10.5 (3.6)	10.6 (3.9)
5–12 years old	337 (66.1)	132 (72.1)	205 (62.7)	–	–	243 (67.9)	93 (66.4)
13–18 years old	173 (33.9)	51 (27.9)	122 (37.3)	–	–	115 (32.1)	47 (33.6)
Child Gender							
Male	358 (70.2)	133 (72.7)	225 (68.8)	243 (72.1)	115 (66.5)	–	–
Female	140 (27.5)	48 (26.2)	92 28.1)	93 (27.6)	47 (27.2)	–	–
Transgender female	5 (1.0)	0 (0.0)	5 (1.5)	1 (0.3)	4 (2.3)	–	–
Transgender male	4 (0.8)	1 (0.6)	3 (0.9)	0 (0.0)	4 (2.3)	–	–
Other	2 (0.4)	0 (0.0)	2 (0.6)	0 (0.0)	2 (1.2)	–	–
Prefer not to answer	1 (0.2)	1 (0.6)	0 (0.0)	0 (0.0)	1 (0.6)	–	–
Report type							
Proxy report	183 (35.9)	–	–	132 (39.2)	51 (29.5)	133 (37.2)	48 (34.3)
Self-report	327 (64.1)	–	–	205 (60.8)	122 (70.5)	225 (68.9)	92 (65.7)

### Acceptability

Overall, the highest proportion of respondents reporting the instrument either ‘easy’ or ‘very easy’ to complete was the EQ-5D-Y-5L with 63.1%. The KIDSCREEN-27 had the lowest proportion at 54.7% ‘easy’ or ‘very easy’ to complete (see Supplementary Figure S1).

Within subgroups, across all instruments, the proportion who reported the instrument either ‘somewhat easy’ or ‘very easy’ was slightly higher for proxy (range = 55.8–66.2%) than self-complete (range = 54.1–61.4%); slightly higher for the 5–12 years old group (range = 57.0–66.5%) than the 13–18 years old group (range = 50.3–58.2%); and slightly higher for males (range = 58.1–66.7%) than females (range = 47.9–56.4%) (see Supplementary Table S2 and Figures S2–S4).

### Feasibility

In the total sample, the median time to complete each of the instruments ranged from 32.1 s (EQ-5D-Y-5L) to 123.5 s (KIDSCREEN-27). Within subgroups there was little variation in median completion times between proxy and self-complete, children 5–12 years old and 13–18 years old, or between male and female (see Supplementary Table S3), with times varying by a maximum of 10 s between subgroups on any instrument.

### Test-retest reliability

The PedsQL, EQ-5D-Y-5L and EQ-5D-Y-3L showed moderate test-retest reliability in the total sample, with confidence intervals ranging from poor to moderate reliability (ICC = 0.56, 95% CI 0.39–0.69); ICC = 0.60, 95% CI 0.47–0.71; ICC = 0.58, 95% CI 0.43–0.69, respectively). The CHU9D showed poor agreement in the total sample, though similar to other instruments, confidence intervals ranged from poor to moderate (ICC = 0.42, 95% CI 0.22–0.58) (see Supplementary Table S4).

Low sample size hindered subgroup comparisons (see Supplementary Table S4).

### Floor and ceiling effects

Within the total sample there were no ceiling effects observed for any of the instruments. Within subgroups, ceiling effects were detected only for the EQ-5D-Y-3L in subgroups of 13–18 year olds (19.1%), in males (15.6%), and self-report (15.0%) (see Supplementary Table S5).

No floor effects were observed for any instrument in the total sample or any subgroups (see Supplementary Table S6).

### Convergent validity

In the total sample there was a strong correlation (> 0.5) between the PedsQL and each of the other HRQoL instruments, ranging from 0.53 (for the KIDSCREEN-27) to 0.63–0.66 (EQ-5D-Y-5L, EQ-5D-Y-3L and CHU9D) (see Supplementary Table S7). The same pattern of results was observed in all subgroups. All observed correlations were as hypothesised, though with a stronger correlation than predicted for the KIDSCREEN-27.

### Known-groups validity

In the total sample, in the test for known groups differences based on ASD severity (children attending mainstream vs. specialist school), significant differences (all *p* < 0.018) were detected with small effect sizes for the PedsQL, EQ-5D-Y-3L and CHU9D (Cohen’s *d* between 0.32 and 0.41). Significant and moderate differences were detected for the EQ-5D-Y-5L (Cohen’s *d =* 0.51, *p* < 0.001). Known groups for ASD severity were not detected using the KIDSCREEN-27 (*p* = 0.598) (see Supplementary Table S8).

For known groups based on SHCN (children without vs. with a special healthcare need), the significant differences with moderate effect sizes were observed for the PedsQL (Cohen’s *d* = 0.64, *p* < 0.001) and CHU9D (Cohen’s *d* = 0.54, *p* < 0.001). Significant differences with small effect sizes were observed for the EQ-5D-Y-5L, EQ-5D-Y-3L, and the KIDSCREEN-27 (Cohen’s *d* between 0.31 and 0.46, all *p* < 0.005) (see Supplementary Table S8).

For known groups based on SDQ symptom thresholds (normal vs. ‘abnormal’ or ‘of concern’ behavioural difficulties as per SDQ), all instruments showed significant differences with strong effect sizes, the highest being the PedsQL (Cohen’s *d* = 1.24) (see Supplementary Table S8).

A similar pattern of results was observed across all subgroups of interest (see Supplementary Table S9). However, a number of subgroups contained small sample sizes and results need to be interpreted with caution.

### Sensitivity analyses

Results from sensitivity analyses utilising utility scores for the EQ-5D-Y-3L and CHU9D were largely unchanged. Test-retest reliability in the CHU9D proxy report subgroup reduced from moderate (ICC = 0.51, 95% CI 0.21–0.71) to poor (ICC = 0.45, 95% CI 0.17–0.67) but otherwise reliability remained consistent (see Supplementary Table S11). Within the known-groups analysis using utility scores, no change in effect size estimate interpretations were noted when compared to analysis utilising total score/LSS (see Supplementary Table S11).

### Summary of instrument performance

A summary of each instrument’s performance in the total sample and within sub-groups is provided in Table 3.

**Table 3:** Summary of instrument performance by total sample, report type, child age and child gender

	Total sample	Report type		Child age		Child gender	
		Proxy	Self	5–12 years	13–18 years	Male	Female
*Test-retest Reliability* ^a^							
PedsQL	**ü**	?	**ü**	**x**	?	**ü**	?
EQ-5D-Y-5L	**ü**	?	**x**	**ü**	?	**ü**	?
EQ-5D-Y-3L	**ü**	?	**ü**	**x**	?	**ü**	?
CHU9D	**x**	?	**x**	**x**	?	**x**	?
KIDSCREEN-27	N/A	N/A	N/A	N/A	N/A	N/A	N/A
*Ceiling effect* ^b^							
PedsQL	**ü**	**ü**	**ü**	**ü**	**ü**	**ü**	**ü**
EQ-5D-Y-5L	**ü**	**ü**	**ü**	**ü**	**ü**	**ü**	**ü**
EQ-5D-Y-3L	**ü**	**ü**	**x**	**ü**	**x**	**x**	**ü**
CHU9D	**ü**	**ü**	**ü**	**ü**	**ü**	**ü**	**ü**
KIDSCREEN-27	**ü**	**ü**	**ü**	**ü**	**ü**	**ü**	**ü**
*Convergent validity* ^c^							
EQ-5D-Y-5L	**ü**	**ü**	**ü**	**ü**	**ü**	**ü**	**ü**
EQ-5D-Y-3L	**ü**	**ü**	**ü**	**ü**	**ü**	**ü**	**ü**
CHU9D	**ü**	**ü**	**ü**	**ü**	**ü**	**ü**	**ü**
KIDSCREEN-27	**ü**	**ü**	**ü**	**ü**	**ü**	**ü**	**ü**
*Known-group – ASD severity (mainstream vs. specialist school)* ^d^							
PedsQL	**x**	?	?	?	?	?	?
EQ-5D-Y-5L	**ü**	?	?	?	?	?	?
EQ-5D-Y-3L	**x**	?	?	?	?	?	?
CHU9D	**x**	?	?	?	?	?	?
KIDSCREEN-27	**x**	?	?	?	?	?	?
*Known-group – Special Healthcare Needs (Y/N)* ^d^							
PedsQL	**ü**	?	**x**	**ü**	?	**ü**	?
EQ-5D-Y-5L	**x**	?	**x**	**x**	?	**ü**	?
EQ-5D-Y-3L	**x**	?	**x**	**x**	?	**ü**	?
CHU9D	**ü**	?	**x**	**x**	?	**ü**	?
KIDSCREEN-27	**x**	?	**x**	**x**	?	**x**	?
*Known-group – SDQ scores (normal vs. ‘abnormal’/‘of concern’ behavioral difficulties)* ^d^							
PedsQL	**ü**	?	**ü**	**ü**	**ü**	**ü**	?
EQ-5D-Y-5L	**ü**	?	**ü**	**ü**	**ü**	**ü**	?
EQ-5D-Y-3L	**ü**	?	**ü**	**ü**	**ü**	**ü**	?
CHU9D	**ü**	?	**ü**	**ü**	**ü**	**ü**	?
KIDSCREEN-27	**ü**	?	**ü**	**ü**	**ü**	**ü**	?

^a^Test retest reliability - ICC < 0.5 (poor), 0.50–0.74 (moderate), 0.75–0.90 (good/excellent);

^b^ Ceiling effect - <15%,$\:\ge\:$15% of respondents reporting best state on all instrument items;

^c^ Convergent validity – Correlation 0.1–0.29 (weak), 0.3–0.49 (moderate), > 0.5 (strong);

^d^ Known-groups validity – Cohen’s *d* ES < 0.5 (small), ES 0.5–0.79 (moderate), ES > 0.8 (large).

N/A Not available as KIDSCREEN-27 not repeated; **?** Not reported due to small sample sizes.

## Discussion

This study examined the comparative psychometric performance of the PedsQL, EQ-5D-Y-5L, EQ-5D-Y-3L, CHU9D and KIDSCREEN-27 in 510 autistic children aged 5–18 years. This study has generated new evidence about the use of these instruments in this population. Overall, the PedsQL and EQ-5D-Y-5L demonstrated the strongest performance across the total sample and within the subgroups of interest. Other instruments tended to perform less well either within a particular psychometric property or subgroup. For example, although the CHU9D showed no ceiling effects, good convergent validity, was able to differentiate between SHCN and SDQ known groups; it demonstrated poor test-retest reliability. The KIDSCREEN-27 had no ceiling effects and showed good convergent validity but showed poorer performance on acceptability.

All instruments demonstrated similarly good acceptability (ease of completing) and feasibility (time to complete). Interestingly, between 55 and 63% of participants in this study reported the instruments as easy to complete, which is lower than in other samples of the P-MIC study where this was over 70% [17, 29], suggesting the instruments are relatively more difficult to complete for autistic children and their caregivers. In this study, only the PedsQL, EQ-5D-Y-5L and EQ-5D-Y-3L demonstrated test-retest reliability in autistic children, with the CHU9D demonstrating poor reliability. Comparatively, in a large sample of Australian children aged 5–18 years of age, the CHU9D, alongside the PedsQL, EQ-5D-Y-5L and EQ-5D-Y-3L was found to be reliable, indicating the CHU9D is less reliable in autistic children [19]. We expected the EQ-5D-Y-5L, EQ-5D-Y-3L, CHU9D to all be strongly correlated with the PedsQL, and all total score results and subgroups were consistent with this expectation. The strong convergent validity of the CHU9D, compared to the PedsQL, contrasted with results reported in the study by Raghunandan et al. where Spearman’s correlations were weak [15]. These weak correlations may be due to the Raghunandan et al. study having only compared proxy completed PedsQL to self-completed CHU9D [15].

Known-groups validity is an indicator that the instrument is detecting differences between groups of children expected to have different HRQoL. All instruments in this study demonstrated the ability to differentiate between children with and without behavioral difficulties (as per the SDQ) with large effect sizes. The PedsQL and CHU9D were additionally able to detect differences between children with and without special healthcare needs, and the EQ-5D-Y-5L was additionally able to differentiate between children attending mainstream versus specialist schools (a proxy for ASD severity). These differences in known-groups validity results likely reflect more heavily on limitations in the choice of the known-groups definition rather than the performance ability of the instruments. For example, poor performance of the instruments on autism severity may suggest that school attendance as a proxy for autism severity may not be the most accurate definition. Autistic children at mainstream schools can struggle, facing difficulties in the schooling environments designed for neurotypical children and may also have poor HRQoL [37]. Conversely – children attending special schools may thrive in a tailored environment that better caters for their needs and have higher HRQoL.

When choosing instruments for use in a clinical context, how the instrument is intended to be used may also be a consideration with some measures of instrument validity more important in some contexts. For example, if the instrument use is to provide clinically valuable information not just scores, instruments with more items may be preferred. This may be lacking in some shorter survey instruments with only 5 items – such as the EQ-5D-Y-5L or EQ-5D-Y-3L – where constructs are ‘compressed’. Further, whilst the KIDSCREEN-27 was rated as more difficult to complete and took longer to complete than other (shorter) instruments, it has the distinct advantage of being framed using positive language consistent with strength-based approaches preferred by some clinicians when working with autistic children and young people [38, 39]. Additionally, as we saw some children aged 7 and above in this study relying on proxy-report rather than self-report, future research may explore the use of interviewer-administered instruments to facilitate self-report, thereby supporting accurate representation of the child’s own perspective. This may be particularly beneficial for younger children or those still developing literacy skills. These considerations did not form part of our acceptability assessment in this study but may be a strong consideration in choice of instrument. Some uses may show a strong preference for a shorter instrument which was measured in this study (for example the EQ-5D-5L took median 32.1 s to complete with 5 items compared to the PedsQL which took median 95.6 s with 23 items).

In contrast to clinical settings, the instruments which may be preferred in a health economic and policy setting are likely to be the preference-accompanied instruments (i.e. the EQ-5D-Y-3L and CHU9D, and, soon, the PedsQL which has a value set in development [40]). These instruments are preferred because they allow the calculation of an overall utility score, which can be used in the calculation of and quality-adjusted life years (QALYs) used to inform priority setting decisions. We were able to repeat psychometric analyses for utility scores of the EQ-5D-Y-3L and CHU9D which showed comparable results to the LSS. QALYs are used by health policy decision-makers around the world to enable a comparison across treatments and interventions which have widely varying contexts, outcomes and impacts [41]. Thus, the choice of instrument may differ depending on the context and intended use of the instrument.

This is the first study exploring psychometric properties across a range of instruments, in the largest sample of autistic children, extending down to age 5 years old, using self-report and proxy. To date this provides the best evidence on the comparative acceptability, feasibility, reliability, and validity of generic HRQoL instruments in a paediatric autistic population. This study has several limitations. The sample was recruited via an online panel, which may have greater potential for sampling bias, self-selection into the survey by participants, inattention by those completing the survey, and the inability to verify if self-report occurred. However, mitigations were put into place to minimise the effects of this within the P-MIC study sample, including quality checks and minimum quality eligibility criteria [20]. Additionally, whilst the sample size of autistic children and young people was adequate for much of the analysis, results for a number of subgroup analysis, particularly within reliability, were unable to be dependably reported due to inadequate sample sizes [36]. We defined autism by parent report of the child being diagnosed with autism spectrum disorder by a health professional but we did not confirm the diagnosis with a multi-disciplinary assessment as per Australian guideline recommendations [42]. Finally, responsiveness to changes in health status is an important psychometric property of HRQoL instruments however, this was unable to be evaluated due to too few children indicating a change in health state between the initial and follow-up surveys.

## Conclusion

All instruments performed well in this study, however, the PedsQL and EQ-5D-Y-5L demonstrated the strongest performance overall and may be a suitable choices for those wishing to measure the HRQoL of autistic children. More evidence on the responsiveness of measures in autistic children is needed and should be the focus of future research.

## Supplementary Information

Below is the link to the electronic supplementary material.

Supplementary Material 1

## Data Availability

Deidentified data may be made available upon reasonable request.
