# Supplementary material for: Measuring quality of life in autistic children and young people: comparing the performance of common generic health related quality of life instruments
Source: Qual Life Res. 2026 Jun 5;35(7):163. doi: 10.1007/s11136-026-04246-4 (PMC13241415; doi:10.1007/s11136-026-04246-4)
Supplement: Supplementary file 1 — Supplementary Material 1 [file 11136_2026_4246_MOESM1_ESM.pdf]

Supplementary Material

Supplementary Table S1: P-MIC study ASD sample, Instruments completed at Initial and Follow-up surveys

Instrument	Initial survey	Follow-up survey
**Demographic and non-HRQoL instruments**		
Demographic Information	✓	
SDQ	✓	
Change in Health State		✓
**HRQoL instruments**		
PedsQL	✓	✓
EQ-5D-Y-5L	✓	✓
EQ-5D-Y-3L	✓	✓
CHU9D	✓	✓
KIDSCREEN-27	✓	

Supplementary Table S2: Acceptability of all instruments by total sample, report type, child age and child gender

Instrument	Total Sample n (%)	Report type		Child age		Child gender	
		Proxy n (%)	Self n (%)	5-12 years n (%)	13-18 years n (%)	Proxy n (%)	Self n (%)
**PedsQL**							
Very difficult	13 (2.5)	4 (2.2)	9 (2.8)	7 (2.1)	6 (3.5)	10 (2.8)	2 (1.4)
Difficult	71 (13.9)	26 (14.2)	45 (13.8)	45 (13.4)	26 (15.0)	46 (12.8)	24 (17.1)
Neutral	127 (24.9)	42 (23.0)	85 (26.0)	76 (22.6)	51 (29.5)	84 (23.5)	37 (26.4)
Easy	133 (26.1)	40 (21.9)	93 (28.4)	81 (24.0)	52 (30.1)	91 (25.4)	40 (28.6)
Very easy	166 (32.5)	71 (38.8)	95 (29.1)	128 (38.0)	38 (22.0)	127 (35.5)	37 (26.4)
**EQ-5D-Y-5L**							
Very difficult	14 (2.7)	4 (2.2)	10 (3.1)	6 (1.8)	8 (4.6)	10 (2.8)	2 (1.4)
Difficult	46 (9.0)	16 (8.7)	30 (9.2)	32 (9.5)	14 (8.1)	30 (8.4)	15 (10.7)
Neutral	128 (25.1)	42 (23.0)	86 (26.3)	75 (22.3)	53 (30.6)	79 (22.1)	44 (31.4)
Easy	119 (23.3)	42 (23.0)	77 (23.5)	72 (21.4)	47 (27.2)	85 (23.7)	32 (22.9)
Very easy	203 (39.8)	79 (43.2)	124 (37.9)	152 (45.1)	51 (29.5)	154 (43.0)	47 (33.6)
**EQ-5D-Y-3L**							
Very difficult	11 (2.2)	4 (2.2)	7 (2.1)	8 (2.4)	3 (1.7)	7 (2.0)	2 (1.4)
Difficult	64 (12.5)	25 (13.7)	39 (11.9)	38 (11.3)	26 (15.0)	40 (11.2)	22 (15.7)
Neutral	129 (25.3)	44 (24.0)	85 (26.0)	81 (24.0)	48 (27.7)	85 (23.7)	40 (28.6)
Easy	130 (25.5)	49 (26.8)	81 (24.8)	72 (21.4)	58 (33.5)	90 (25.1)	38 (27.1)
Very easy	176 (34.5)	61 (33.3)	115 (35.2)	138 (40.9)	38 (22.0)	136 (38.0)	38 (27.1)
**CHU9D**							
Very difficult	15 (2.9)	5 (2.7)	10 (3.1)	10 (3.0)	5 (2.9)	12 (3.4)	1 (0.7)
Difficult	49 (9.6)	19 (10.4)	30 (9.2)	31 (9.2)	18 (10.4)	34 (9.5)	14 (10.0)
Neutral	137 (26.9)	46 (25.1)	91 (27.8)	88 (26.1)	49 (28.3)	86 (24.0)	46 (32.9)
Easy	125 (24.5)	43 (23.5)	82 (25.1)	71 (21.1)	54 (31.2)	88 (24.6)	36 (25.7)

Very easy	184 (36.1)	70 (38.3)	114 (34.9)	137 (40.7)	47 (27.2)	138 (38.5)	43 (30.7)
**KIDSCREEN-27**							
Very difficult	27 (5.3)	9 (4.9)	18 (5.5)	20 (5.9)	7 (4.0)	18 (5.0)	8 (5.7)
Difficult	89 (17.5)	38 (20.8)	51 (15.6)	54 (16.0)	35 (20.2)	60 (16.8)	27 (19.3)
Neutral	115 (22.5)	34 (18.6)	81 (24.8)	71 (21.1)	44 (25.4)	72 (20.1)	38 (27.1)
Easy	125 (24.5)	42 (23.0)	83 (25.4)	74 (22.0)	51 (29.5)	96 (26.8)	27 (19.3)
Very easy	154 (30.2)	60 (32.8)	94 (28.7)	118 (35.0)	36 (20.8)	112 (31.3)	40 (28.6)
	54.7	55.8	54.1	57	50.3	58.2	47.9

Supplementary Figure S1: Acceptability of each instrument (total sample)

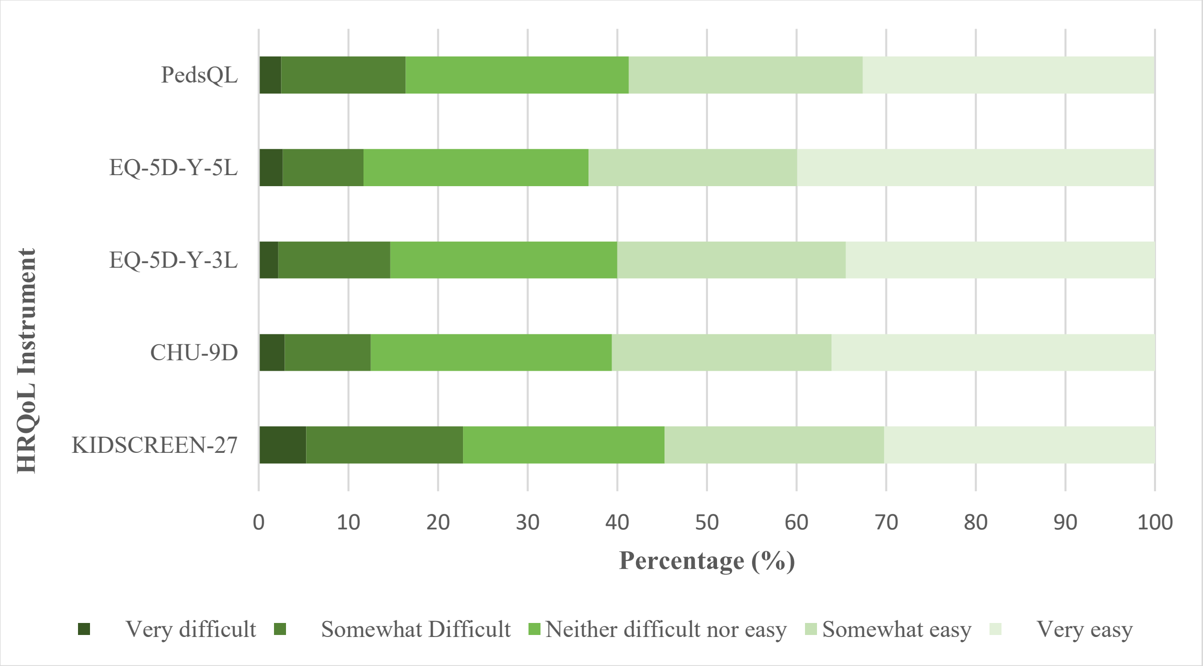

Supplementary Figure S2: Acceptability of each instrument by report type

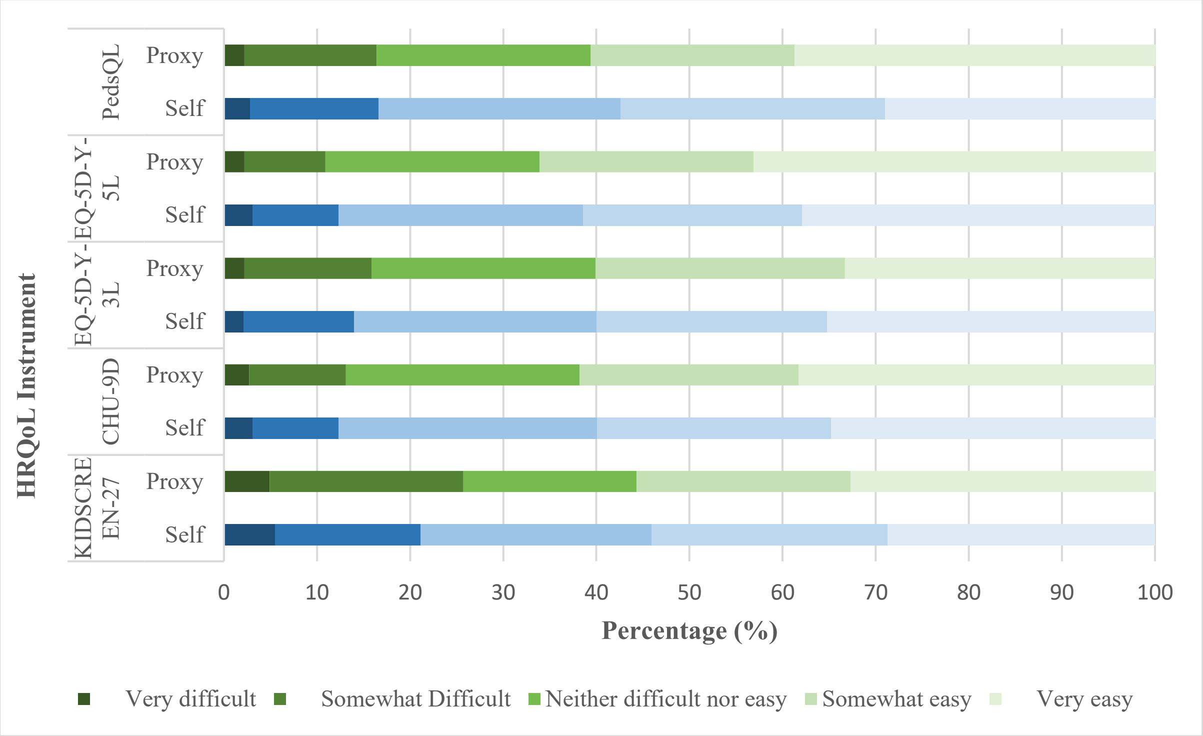

Supplementary Figure S3: Acceptability of each instrument by child age

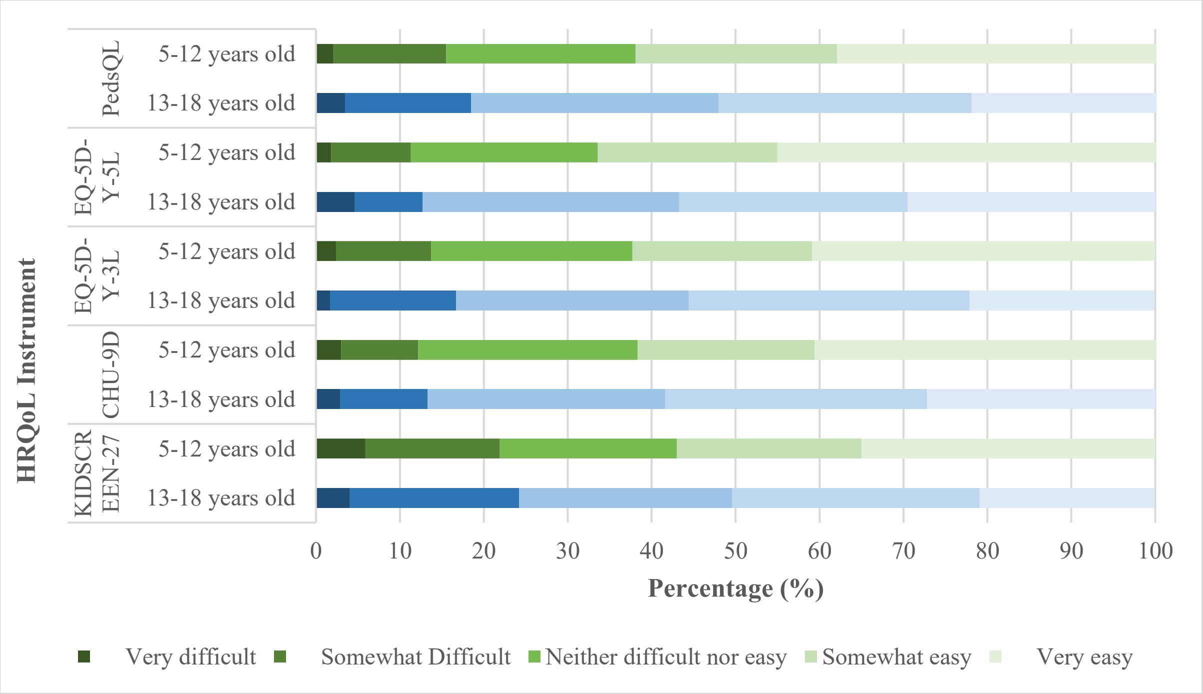

Supplementary Figure S4: Acceptability of each instrument by child gender

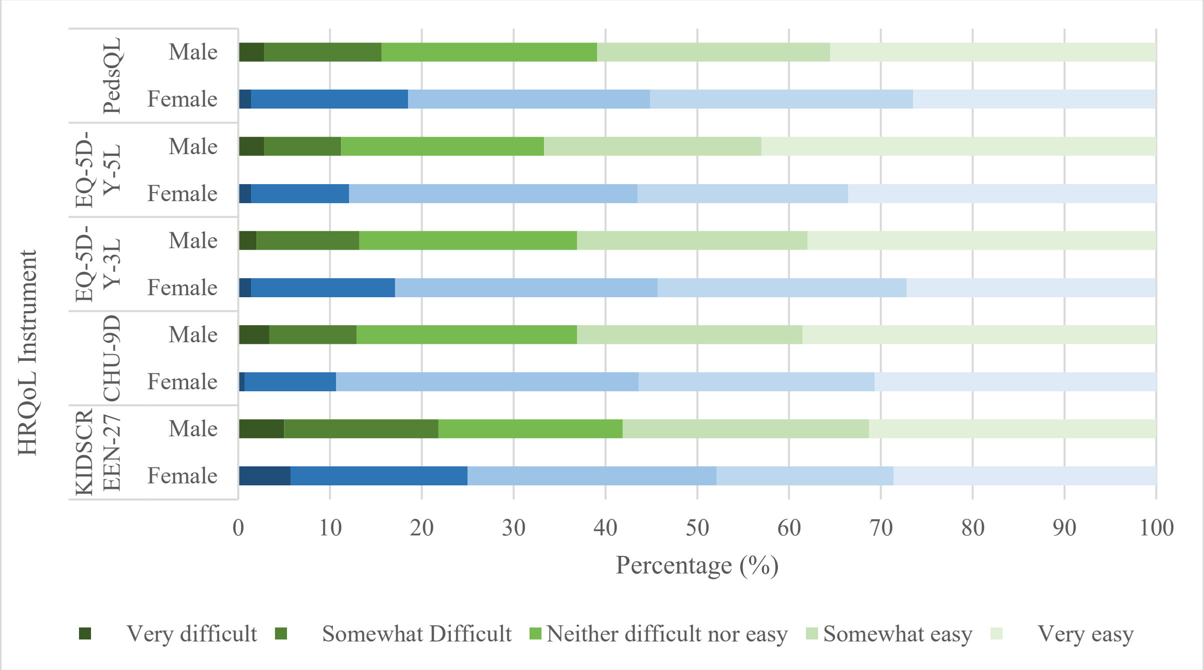

Supplementary Table S3: Time to complete each instrument (seconds) by total sample, report type, child age and child gender

Instrument	Total Sample median (IQR)	Report type		Child age		Child gender	
		Proxy median (IQR)	Self median (IQR)	5-12 years median (IQR)	13-18 years median (IQR)	Proxy median (IQR)	Self median (IQR)
**PedsQL**	95.6 (73.5, 127.2)	93.8 (72.4, 124.3)	96.0 (74.6, 130.7)	95.5 (72.8, 130.0)	96.0 (75.2, 125.5)	95.8 (72.3, 127.2)	95.2 (78.1, 130.2)
**EQ-5D-Y-5L**	32.1 (23.6, 46.1)	31.9 (22.1, 42.6)	32.9 (24.0, 48.6)	33.3 (23.9, 47.6)	29.9 (22.9, 43.9)	32.6 (23.7, 47.7)	30.9 (23.0, 43.1)
**EQ-5D-Y-3L**	46.7 (35.8, 64.2)	46.6 (35.7, 64.2)	46.8 (35.9, 64.6)	46.8 (35.7, 67.1)	46.6 (36.7, 58.8)	47.0 (35.8, 63.9)	45.6 (35.8, 65.6)
**CHU9D**	58.1 (44.6, 79.2)	57.4 (44.7, 76.3)	58.9 (44.2, 82.0)	59.0 (44.7, 82.0)	57.9 (44.3, 76.4)	58.8 (44.9, 80.6)	57.7 (43.5, 75.8)
**KIDSCREEN-27**	123.5 (96.3, 162.1)	129.1 (101.6, 171.3)	119.5 (93.97, 156.1)	125.3 (94.9, 159.2)	120.7 (102.9, 165.4)	124.2 (96.3, 161.3)	123.5 (97.5, 168.4)

Supplementary Table S4: Intra-class correlation coefficients (ICC) of each instrument by total sample, report type, child age, and child gender

Instrument	Total sample	Report type		Child age		Child gender	
		Proxy	Self	5-12 years	13-18 years	Male	Female
	ICC (95% CI) n=106	ICC (95% CI) **n=39**	ICC (95% CI) n=67	ICC (95% CI) n=75	ICC (95% CI) **n=31**	ICC (95% CI) n=81	ICC (95% CI) **n=24**
**PedsQL**	0.56 (0.39, 0.69)	0.32 (0.02, 0.57)	0.72 (0.58, 0.82)	0.43 (0.20, 0.61)	0.88 (0.77, 0.94)	0.60 (0.43, 0.73)	0.50 (0.14, 0.74)
**EQ-5D-Y-5L**	0.60 (0.47, 0.71)	0.76 (0.59, 0.87)	0.49 (0.28, 0.66)	0.51 (0.32, 0.66)	0.75 (0.55, 0.87)	0.62 (0.46, 0.74)	0.56 (0.21, 0.79)
**EQ-5D-Y-3L**	0.58 (0.43, 0.69)	0.55 (0.28, 0.73)	0.60 (0.42, 0.73)	0.44 (0.24, 0.61)	0.85 (0.71, 0.92)	0.53 (0.36, 0.67)	0.63 (0.31, 0.82)
**CHU9D**	0.42 (0.22, 0.58)	0.51 (0.21, 0.71)	0.37 (0.14, 0.56)	0.24 (0.03, 0.44)	0.73 (0.51, 0.86)	0.36 (0.14, 0.54)	0.68 (0.39, 0.84)

Inadequate sample size n=<30, Doubtful Sample Size n=30-49 based on the COSMIN guidelines[38], Reliability based on Koo et al 2016: Poor reliability <0.5 (white); Moderate reliability 0.50-0.74 (light blue); Good reliability 0.75-0.90 (medium blue); Excellent reliability >0.90 (Dark blue)

Supplementary Table S5: Ceiling effect of each instrument by total sample, report type, child age, and child gender

Instrument	Total ASD Sample n (%)	Report type		Child age		Child gender	
		Proxy n (%)	Self n (%)	5-12 years n (%)	13-18 years n (%)	Male n (%)	Female n (%)
**PedsQL**	1 (0.2)	0 (0.0)	1 (0.3)	1 (0.3)	0 (0.0)	1 (0.3)	0 (0.0)
**EQ-5D-Y-5L**	52 (10.2)	16 (8.7)	36 (11.0)	28 (8.3)	24 (13.9)	42 (11.7)	9 (6.4)
**EQ-5D-Y-3L**	72 (14.1)	23 (12.6)	49 (15.0)	39 (11.6)	33 (19.1)	56 (15.6)	14 (10.0)
**CHU9D**	7 (1.4)	2 (1.1)	5 (1.5)	4 (1.2)	3 (1.7)	6 (1.7)	1 (0.7)
**Kidscreen-27**	0 (0.0)	0 (0.0)	0 (0.0)	0 (0.0)	0 (0.0)	0 (0.0)	0 (0.0)

Flag for ceiling effect - ≥15% of respondents reporting best state on all instrument items (medium blue)

Supplementary Table S6: Floor Effect of all instruments by total sample, report type, child age and child gender

Instrument	Total Sample n (%)	Report type		Child age		Child gender	
		Proxy n (%)	Self n (%)	5-12 years n (%)	13-18 years n (%)	Male n (%)	Female n (%)
**PedsQL**	0 (0.0)	0 (0.0)	0 (0.0)	0 (0.0)	0 (0.0)	0 (0.0)	0 (0.0)
**EQ-5D-Y-5L**	1 (0.2)	1 (0.5)	0 (0.0)	1 (0.3)	0 (0.0)	1 (0.3)	0 (0.0)
**EQ-5D-Y-3L**	2 (0.4)	1 (0.5)	1 (0.3)	1 (0.3)	1 (0.6)	2 (0.6)	0 (0.0)
**CHU9D**	1 (0.2)	1 (0.5)	0 (0.0)	1 (0.3)	0 (0.0)	1 (0.3)	0 (0.0)
**Kidscreen-27**	1 (0.2)	0 (0.0)	0 (0.0)	1 (0.3)	0 (0.0)	1 (0.3)	0 (0.0)

Flag for ceiling effect - ≥15% of respondents reporting worst state on all instrument items (medium blue)

Supplementary Table S7: Spearman correlation of PedsQL and each instrument by total sample, report type, child age, and child gender

Instrument	Total sample	Report type		Child age		Child gender	
		Proxy	Self	5-12 years	13-18 years	Male	Female
**EQ-5D-Y-5L**	-0.66	-0.60	-0.69	-0.67	-0.67	-0.68	-0.59
**EQ-5D-Y-3L**	-0.65	-0.61	-0.65	-0.67	-0.65	-0.67	-0.62
**CHU9D**	-0.63	-0.56	-0.67	-0.63	-0.64	-0.64	-0.59
**Kidscreen-27**	0.53	0.46	0.58	0.51	0.50	0.52	0.52

Correlation 0.1-0.29 (white) is considered weak, 0.3-0.49 (light blue) moderate, and ≥0.5 (medium blue) strong

Supplementary Table S8: Known-group analysis of instruments within the total sample

Instrument	High ASD Severity				Special Health Care Needs				Abnormal SDQ Total Score			
	No N=395 M (SD)	Yes N=64 M (SD)	*P*-value	Cohen's *d*	No N=115 M (SD)	Yes N=395 M (SD)	*P*-value	Cohen's *d*	No N=149 M (SD)	Yes N=349 M (SD)	*P*-value	Cohen's *d*
**PedsQL**	56.15 (16.26)	50.92 (16.04)	0.017	-0.32	62.50 (14.66)	52.41 (16.14)	<0.001	-0.64	67.22 (13.33)	49.65 (14.57)	<0.001	-1.24
**EQ-5D-Y-5L**	8.75 (3.19)	10.44 (3.78)	<0.001	0.51	7.87 (2.73)	9.41 (3.47)	<0.001	0.46	7.26 (2.53)	9.79 (3.83)	<0.001	0.8
**EQ-5D-Y-3L**	7.37 (1.81)	8.06 (2.17)	0.006	0.37	6.85 (1.61)	7.71 (1.93)	<0.001	0.46	6.46 (1.52)	7.97 (1.86)	<0.001	0.85
**CHU9D**	18.79 (6.49)	21.50 (7.74)	0.003	0.41	16.63 (5.62)	20.19 (6.92)	<0.001	0.54	15.30 (5.17)	21.00 (6.62)	<0.001	0.92
**KIDSCREEN -27**	90.07 (16.66)	88.88 (17.41)	0.598	0.07	93.10 (16.72)	87.92 (16.75)	0.004	0.31	98.46 (14.53)	85.55 (16.26)	<0.001	0.82

Cohen's *d* used for effect size (ES) estimate: Small ES 0.2-0.49 (light blue); Moderate ES 0.5-0.79 (medium blue); Large ES ≥0.8 (Dark blue).

Supplementary Table S9: Known-group analysis of instruments by report type, child age and child gender

Instrument	High ASD Severity				Special Health Care Needs				Abnormal SDQ Total Score			
	No M (SD)	Yes M (SD)	*P*-value	Cohen's *d*	No M (SD)	Yes M (SD)	*P*-value	Cohen's *d*	No M (SD)	Yes M (SD)	*P*-value	Cohen's *d*
**SUBGROUP - REPORT TYPE**												
**Proxy report**												
**Sample size**	n=123	n=38			n=32	n=151			n=38	n=143		
**PedsQL**	54.65 (16.06)	49.46 (14.64)	0.077	-0.33	64.81 (14.80)	49.97 (14.83)	<0.001	-1.00	66.53 (14.43)	48.94 (14.17)	<0.001	-1.24
**EQ-5D-Y-5L**	8.95 (3.39)	10.74 (3.74)	0.006	0.51	7.22 (1.60)	9.92 (3.78)	<0.001	0.77	7.37 (2.47)	10.03 (3.71)	<0.001	0.76
**EQ-5D-Y-3L**	7.53 (1.81)	8.34 (2.12)	0.021	0.43	6.75 (1.34)	7.99 (1.99)	<0.001	0.65	6.42 (1.62)	8.17 (1.87)	<0.001	0.96
**CHU9D**	18.95 (6.55)	20.79 (8.42)	0.16	0.26	14.84 (4.24)	20.47 (7.20)	<0.001	0.83	14.11 (4.28)	20.99 (7.02)	<0.001	1.05
**KIDSCREEN-27**	92.15 (15.95)	90.21 (18.91)	0.53	0.11	100.34 (16.78)	89.09 (15.98)	<0.001	0.70	99.87 (15.35)	88.90 (16.25)	<0.001	0.68
**Self-report**												
**Sample Size**	n=272	n=26			n=83	n=244			n=111	n=206		
**PedsQL**	56.83 (16.33)	53.05 (17.98)	0.26	-0.23	61.62 (14.59)	53.91 (16.75)	<0.001	-0.47	67.46 (12.99)	50.14 (14.85)	<0.001	-1.22
**EQ-5D-Y-5L**	8.65 (3.10)	10.00 (3.87)	0.040	0.42	8.12 (3.03)	9.09 (3.23)	0.016	0.31	7.23 (2.56)	9.62 (3.13)	<0.001	0.81
**EQ-5D-Y-3L**	7.29 (1.80)	7.65 (2.21)	0.34	0.20	6.89 (1.71)	7.54 (1.87)	0.006	0.35	6.48 (1.49)	7.83 (1.85)	<0.001	0.78
**CHU9D**	18.71 (6.48)	22.54 (6.65)	0.004	0.59	17.31 (5.95)	20.02 (6.75)	0.001	0.41	15.71 (5.40)	21.00 (6.34)	<0.001	0.88
**KIDSCREEN-27**	89.12 (16.92)	86.92 (15.10)	0.52	0.13	90.30 (15.92)	87.19 (17.21)	0.15	0.18	97.98 (14.27)	83.22 (15.89)	<0.001	0.96
**SUBGROUP – CHILD AGE**												
**5-12 years old**												
**Sample size**	n=271	n=39			n=80	n=257			n=89	n=247		
**PedsQL**	56.00 (16.25)	52.20 (16.34)	0.17	-0.23	61.26 (14.19)	52.85 (16.39)	<0.001	-0.53	67.72 (13.52)	50.28 (14.64)	<0.001	-1.21
**EQ-5D-Y-5L**	9.01 (3.25)	10.41 (3.97)	0.015	0.42	8.21 (2.89)	9.58 (3.48)	0.002	0.41	7.52 (2.77)	9.85 (3.38)	<0.001	0.72
**EQ-5D-Y-3L**	7.54 (1.81)	7.95 (2.11)	0.20	0.22	7.19 (1.67)	7.79 (1.91)	0.012	0.33	6.60 (1.52)	8.02 (1.84)	<0.001	0.81
**CHU9D**	18.86 (6.29)	22.03 (7.86)	0.005	0.49	17.06 (5.75)	20.22 (6.69)	<0.001	0.49	15.56 (5.14)	20.81 (6.48)	<0.001	0.85
**KIDSCREEN-27**	92.37 (16.96)	91.10 (19.25)	0.67	0.07	93.84 (17.26)	91.04 (17.05)	0.20	0.16	101.80 (14.60)	88.18 (16.46)	<0.001	0.85
**13-18 years old**												
**Sample Size**	n=124	n=25			n=35	n=138			n=60	n=102		
**PedsQL**	56.48 (16.34)	48.91 (15.67)	0.035	-0.47	65.34 (15.51)	51.57 (15.68)	<0.001	-0.88	66.49 (13.11)	48.14 (14.33)	<0.001	-1.32
**EQ-5D-Y-5L**	8.17 (3.00)	10.48 (3.54)	<0.001	0.75	7.09 (2.16)	9.10 (3.43)	0.001	0.63	6.88 (2.09)	9.62 (3.40)	<0.001	0.92
**EQ-5D-Y-3L**	6.99 (1.74)	8.24 (2.28)	0.002	0.68	6.09 (1.17)	7.57 (1.97)	<0.001	0.81	6.27 (1.51)	7.86 (1.91)	<0.001	0.90
**CHU9D**	18.64 (6.93)	20.68 (7.65)	0.19	0.29	15.63 (5.26)	20.14 (7.35)	<0.001	0.65	14.92 (5.25)	21.44 (6.96)	<0.001	1.02
**KIDSCREEN-27**	85.03 (14.85)	85.40 (13.71)	0.91	0.03	91.40 (15.50)	82.11 (14.55)	0.001	0.63	93.52 (13.02)	79.17 (13.87)	<0.001	1.06
**SUBGROUP – CHILD GENDER**												
**Male**												
**Sample size**	n=281	n=44			n=87	n=271			n=131	n=227		
**PedsQL**	56.61 (16.48)	51.85 (17.14)	0.077	-0.29	64.23 (14.60)	52.62 (16.28)	<0.001	-0.73	66.88 (13.33)	48.84 (14.67)	<0.001	-1.27
**EQ-5D-Y-5L**	8.68 (3.18)	10.66 (4.09)	<0.001	0.60	7.56 (2.40)	9.44 (3.51)	<0.001	0.57	7.18 (2.34)	10.03 (3.44)	<0.001	0.92
**EQ-5D-Y-3L**	7.36 (1.87)	8.16 (2.38)	0.012	0.41	6.76 (1.57)	7.73 (2.00)	<0.001	0.51	6.43 (1.49)	8.11 (1.92)	<0.001	0.95
**CHU9D**	18.74 (6.63)	21.41 (8.02)	0.016	0.39	16.09 (5.27)	20.19 (6.98)	<0.001	0.62	15.47 (5.26)	21.35 (6.71)	<0.001	0.95
**KIDSCREEN-27**	90.51 (17.27)	89.57 (17.10)	0.74	0.05	95.16 (16.38)	88.20 (17.19)	<0.001	0.41	97.97 (14.39)	85.22 (17.05)	<0.001	0.79
**Female**												
**Sample size**	n=108	n=17			n=26	n=114			n=18	n=122		
**PedsQL**	55.51 (15.24)	49.36 (14.42)	0.12	-0.41	58.40 (13.43)	52.45 (15.74)	0.077	-0.39	69.75 (13.45)	51.17 (14.31)	<0.001	-1.31
**EQ-5D-Y-5L**	8.81 (3.14)	10.35 (2.83)	0.060	0.50	8.54 (3.35)	9.29 (3.32)	0.30	0.23	7.89 (3.66)	9.34 (3.25)	0.085	0.44
**EQ-5D-Y-3L**	7.42 (1.62)	8.06 (1.52)	0.13	0.40	7.08 (1.70)	7.69 (1.75)	0.11	0.35	6.72 (1.74)	7.70 (1.72)	0.026	0.57
**CHU9D**	18.79 (6.00)	22.06 (7.56)	0.046	0.53	17.88 (5.61)	19.92 (6.67)	0.15	0.31	14.11 (4.43)	20.34 (6.41)	<0.001	1.01
**KIDSCREEN-27**	89.32 (15.17)	89.88 (18.24)	0.89	0.04	87.58 (16.27)	88.33 (15.63)	0.83	0.05	102.06 (15.41)	86.15 (14.72)	<0.001	1.07

Inadequate sample size n=<30, Doubtful Sample Size n=30-49 based on the COSMIN guidelines[38], therefore presented but not analysed; Cohen's *d* used for effect size (ES) estimate: Small ES 0.2-0.49 (light blue); Moderate ES 0.5-0.79 (medium blue); Large ES ≥0.8 (Dark blue).

Supplementary Table S10: Intra-class correlation coefficients (ICC) of each instrument by total sample, report type, child age, and child gender (Utility scores)

Instrument	Total sample	Report type		Child age		Child gender	
		Proxy	Self	5-12 years	13-18 years	Male	Female
	ICC (95% CI) n=106	ICC (95% CI) **n=39**	ICC (95% CI) n=67	ICC (95% CI) n=75	ICC (95% CI) **n=31**	ICC (95% CI) n=81	ICC (95% CI) **n=24**
**EQ-5D-Y-3L**	0.54 (0.39, 0.66)	0.53 (0.26, 0.72)	0.56 (0.37, 0.70)	0.44 (0.24, 0.61)	0.76 (0.56, 0.88)	0.45 (0.26, 0.61)	0.71 (0.44, 0.87)
**CHU9D**	0.41 (0.22, 0.56)	0.45 (0.17, 0.67)	0.38 (0.16, 0.57)	0.28 (0.07, 0.48)	0.69 (0.46, 0.84)	0.38 (0.16, 0.55)	0.51 (0.16, 0.75)

Inadequate sample size n=<30, Doubtful Sample Size n=30-49 based on the COSMIN guidelines[38], Reliability based on Koo et al 2016: Poor reliability <0.5 (white); Moderate reliability 0.50-0.74 (light blue); Good reliability 0.75-0.90 (medium blue); Excellent reliability >0.90 (Dark blue)

Supplementary Table S11: Known-group analysis of EQ-5D-Y-3L and CHU9D within the total sample (Utility scores)

Instrument	High ASD Severity				Special Health Care Needs				Abnormal SDQ Total Score			
	No N=395 M (SD)	Yes N=64 M (SD)	*P*-value	Cohen's *d*	No N=115 M (SD)	Yes N=395 M (SD)	*P*-value	Cohen's *d*	No N=149 M (SD)	Yes N=349 M (SD)	*P*-value	Cohen's *d*
**EQ-5D-Y-3L**	0.72 (0.24)	0.65 (0.30)	0.03	0.30	0.79 (0.20)	0.68 (0.01)	<0.001	0.43	0.83 (0.18)	0.65 (0.26)	<0.001	0.77
**CHU9D**	0.56 (0.23)	0.49 (0.27)	0.02	0.32	0.64 (0.23)	0.52 (0.24)	<0.001	0.50	0.70 (0.21)	0.48 (0.22)	<0.001	0.99

Cohen's *d* used for effect size (ES) estimate: Small ES 0.2-0.49 (light blue); Moderate ES 0.5-0.79 (medium blue); Large ES ≥0.8 (Dark blue).
